# Complete staghorn calculus in polycystic kidney disease: infection is still the cause

**DOI:** 10.1186/1471-2369-14-168

**Published:** 2013-08-01

**Authors:** Zhiguo Mao, Jing Xu, Chaoyang Ye, Dongping Chen, Changlin Mei

**Affiliations:** 1 Kidney Institute of CPLA, Division of Nephrology, Changzheng Hospital, Second Military Medical University, 415 Fengyang Road, Shanghai 200003, China

**Keywords:** Staghorn calculus, Polycystic kidney disease, Urinary tract infection

## Abstract

**Background:**

Kidney stones in patients with autosomal dominant polycystic kidney disease are common, regarded as the consequence of the combination of anatomic abnormality and metabolic risk factors. However, complete staghorn calculus is rare in polycystic kidney disease and predicts a gloomy prognosis of kidney. For general population, recent data showed metabolic factors were the dominant causes for staghorn calculus, but for polycystic kidney disease patients, the cause for staghorn calculus remained elusive.

**Case presentation:**

We report a case of complete staghorm calculus in a polycystic kidney disease patient induced by repeatedly urinary tract infections. This 37-year-old autosomal dominant polycystic kidney disease female with positive family history was admitted in this hospital for repeatedly upper urinary tract infection for 3 years. CT scan revealed the existence of a complete staghorn calculus in her right kidney, while there was no kidney stone 3 years before, and the urinary stone component analysis showed the composition of calculus was magnesium ammonium phosphate.

**Conclusion:**

UTI is an important complication for polycystic kidney disease and will facilitate the formation of staghorn calculi. As staghorn calculi are associated with kidney fibrosis and high long-term renal deterioration rate, prompt control of urinary tract infection in polycystic kidney disease patient will be beneficial in preventing staghorn calculus formation.

## Background

The incidence of kidney stone in patients with autosomal dominant polycystic kidney disease (ADPKD) is approximately 5 to 10 folds of that in the general population [1-3]. The high incidence of nephrolithiasis is thought to be the consequence of the combination of anatomic abnormality and metabolic risk factors in ADPKD patients [4]. However, complete staghorn calculus in ADPKD is rare and the causes of staghorn calculus in ADPKD remains elusive. As existence of staghorn calculus predicts poor prognosis of kidneys [5,6], the causes for staghorn calculus in ADPKD are of interests of nephrologists.

Here we report a case of complete staghorm calculus in an ADPKD patient induced by repeatedly upper urinary tract infections.

## Case presentation

A 37-year-old ADPKD female patient with positive family history was admitted in this hospital for repeatedly urinary tract infection for 3 years. Three years ago, the patient was admitted in community hospital for urinary tract irritation with right flank pain and fever for 2 days, and right upper urinary tract infection was considered. After admission, bilateral enlarged kidneys full of fluid-filled cysts were detected by type-B ultrasound examination, and there were no calculus or crystals in the kidneys detected at that time. ADPKD was diagnosed considering her family history. Since then, she suffered from urinary tract infection every 3–4 months, and urine cultures were negative. Symptoms usually were relieved within a week with antibiotics therapy, but the infections relapsed easily.

**Figure 1:**
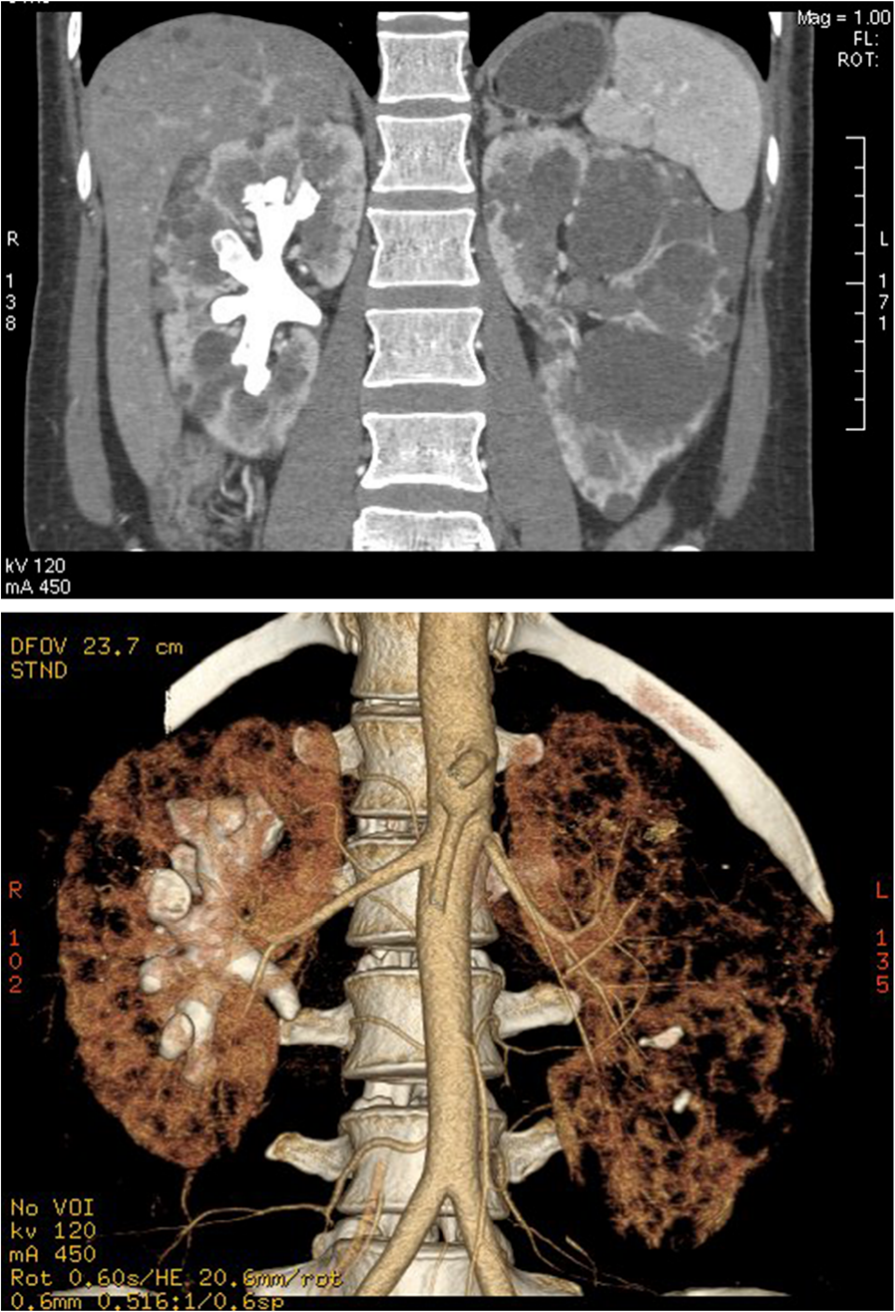
**CT images of a complete staghorn calculus in the right kidney in an ADPKD female.** Above the staghorn calculus and polycystic kidney in the coronal plane; Below the three-dimension reconstructed CT image of calculus and kidney.

One week before admission, this patient’s urinary irritation relapsed with high fever. As oral antibiotics were not responsive, she was admitted in this hospital for further treatment. On admission, her temperature was 39.0°C, blood pressure 110/82 mmHg. Physical examination revealed the bilateral enlarged kidneys that were palpable, tenderness existed in right flank area. Full blood cell count showed white blood cell count 11.1 × 10^9^/l, hemoglobin 12.3 g/dl and platelets 157 × 10^9^/l. A serum biochemical profile showed her renal function was normal with serum creatinine 0.74 mg/dl. Liver function and electrolytes were unremarkable. Urinalysis revealed +3 leukocytes, +2 blood, and +1 albumin. Chest radiology and electrocardiography were reported to be normal. Abdominal CT scan showed enlarged cystic kidneys and a complete staghorn calculus in the right kidney with no obvious signs of urinary tract obstruction (Figure 1). Wide spectrum antibiotics was administered intravenously and the symptoms relieved in 3 days.

Percutaneous nephrolithotomy (PNL) was suggested,but the patient refused surgery as she felt well after the symptom relieved and worried about the surgical risks. To reduce the urinary tract infection and calculus formation, big volume of water intake was suggested to keep urinary output more than 2 L a day.

One month after discharge, the patient reported a “tiny stone” passed with urine, and the urinary stone component analysis showed the composition of calculus was magnesium ammonium phosphate. Considering the calculus component and the rapid formation of the staghorn calculus accompanied by the repeated upper urinary tract infections, the main cause of the staghorn calculus was determined as the repeated upper urinary tract infection in ADPKD.

## Conclusions

The incidence of kidney calculus in ADPKD patients ranges from 8% to 36% [1-3], much higher than in general population, while ADPKD with staghorn calculus is rare. In our center data, there is only one case with complete staghorn kidney calculus among 412 regularly followed ADPKD patients (221 male and 191 female) in the past twelve years.

Calcium oxalate and/or uric acid are the most components of kidney stone in ADPKD, although the percentage of metabolic disturbances was not higher in ADPKD patients with kidney calculus than without [2]. Patients with a larger kidney volume predominant cyst size are at a higher risk of stone formation [2,4]. Numerous expanding kidney cysts distort the intrarenal calyceal system, which leads to urinary stasis and delayed washout of urinary crystals. Hypocitruria, aciduria and low urinary magnesium are common metabolic defects in ADPKD, and these factors facillate the formation and aggregation urinary crystal.

There are no generally accepted causes for the formation of staghorn calculi in ADPKD. In general population, staghorn calculi were traditionally believed to be synonymous with infection stone, and secondary to of UTI [7]. However, recent data challenged this traditional opinion. In a retrospective analysis with 52 kidneys with complete staghorn calculi, 56% of the kidney stones were metabolic and 44% were infection stones [8]. The rising percentage of metabolic stones were also observed in other urinary stone cases [9,10].

In this case of ADPKD patient, the medical history and kidney stone component analysis supported that this case was a typical UTI induced complete staghorn calculus. This case showed in ADPKD patients, UTI was an important cause of staghorn calculus. As UTI is very common in ADPKD with 30%-50% patients experiencing an episode in their lifetime [11,12], and staghorn calculi was associated with kidney fibrosis [5] and high long term renal deterioration rate [6], prompt control of UTI in ADPKD patient will be beneficial in preventing staghorn calculus formation.

For the treatment of staghorn calculi, PNL was recommended as the first therapy for most patients by AUA guideline in 2005 [7], while shock wave lithotripsy (SWL) monotherapy and open surgery are only indicated on few conditions [13]. In ADPKD patients, the principles of management of kidney stones are the same as in patients with normal kidneys, however PNL can be challenging because of the distortion of kidney anatomy by cysts compression and the cysts can come in the way of puncture and need to be aspirated before procedure [1,14-16]. As reports on surgical treatment for nephrolithisasis in ADPKD patients are limited, it is reasonable to choose suitable therapeutic modality based on experience and expertise of each center. After removal of kidney stones, risk factors evaluation and control should always be considered to reduce the frequency of recurrent stone disease in such patients.

## Consent

Written informed consent was obtained from the patient for publication of this Case report and any accompanying images. A copy of the written consent is available for review by the Editor of this journal.

## Abbreviations

ADPKD: Autosomal dominant polycystic kidney disease; UTI: Urinary tract infection.

## Competing interests

The authors declare that they have no competing interests.

## Authors’ contributions

ZM, CY and JX treated the patients and collected the data, ZM and DC wrote the manuscript. CM designed the study and helped to draft the manuscript. All authors read and approved the final manuscript.

## Pre-publication history

The pre-publication history for this paper can be accessed here:

http://www.biomedcentral.com/1471-2369/14/168/prepub
